# Supplementary material for: Antimicrobial Resistance, Virulence Gene Profiling, and Spa Typing of Staphylococcus aureus Isolated from Retail Chicken Meat in Alabama, USA
Source: Pathogens. 2025 Jan 22;14(2):107. doi: 10.3390/pathogens14020107 (PMC11858072; doi:10.3390/pathogens14020107)
Supplement: Supplementary file 1 [file pathogens-14-00107-s001.zip › Supplementary Table S1.pdf]

Table S1. Summary of the number of Samples and *Staphylococcus aureus* (*S. aureus*) isolates recovered from the various chicken parts

Location	Part	No. of Samples	No. Positive	No. Negative	Sample Part Positive (%)	Sample Part Negative (%)
A	Thigh	12	6	6	50.0	50.0
	Gizzard	12	4	8	33.3	66.7
	Liver	12	2	10	16.7	83.3
	Wing	14	1	13	7.1	92.9
	Total	50	13	37	26	74
B	Thigh	13	4	9	30.8	69.2
	Gizzard	13	0	13	0.0	100.0
	Liver	12	1	11	8.3	91.7
	Wing	12	2	10	16.7	83.3
	Total	50	7	43	14	28
C	Thigh	14	1	13	7.1	92.9
	Gizzard	12	1	11	8.3	91.7
	Liver	12	1	11	8.3	91.7
	Wing	12	3	9	25.0	75.0
	Total	50	6	44	12	24
D	Thigh	13	1	12	7.7	92.3
	Gizzard	13	0	13	0.0	100.0
	Liver	12	0	12	0.0	100.0
	Wing	12	5	7	41.7	58.3
	Total	50	6	44	12.0	88.0
	**All total**	**200**	**32**	**168**	**16.0%**	**84.0%**
