# Supplementary figures and images for: Antimicrobial Resistance, Virulence Gene Profiling, and Spa Typing of Staphylococcus aureus Isolated from Retail Chicken Meat in Alabama, USA
Source: Pathogens. 2025 Jan 22;14(2):107. doi: 10.3390/pathogens14020107 (PMC11858072; doi:10.3390/pathogens14020107)

## Slide 1
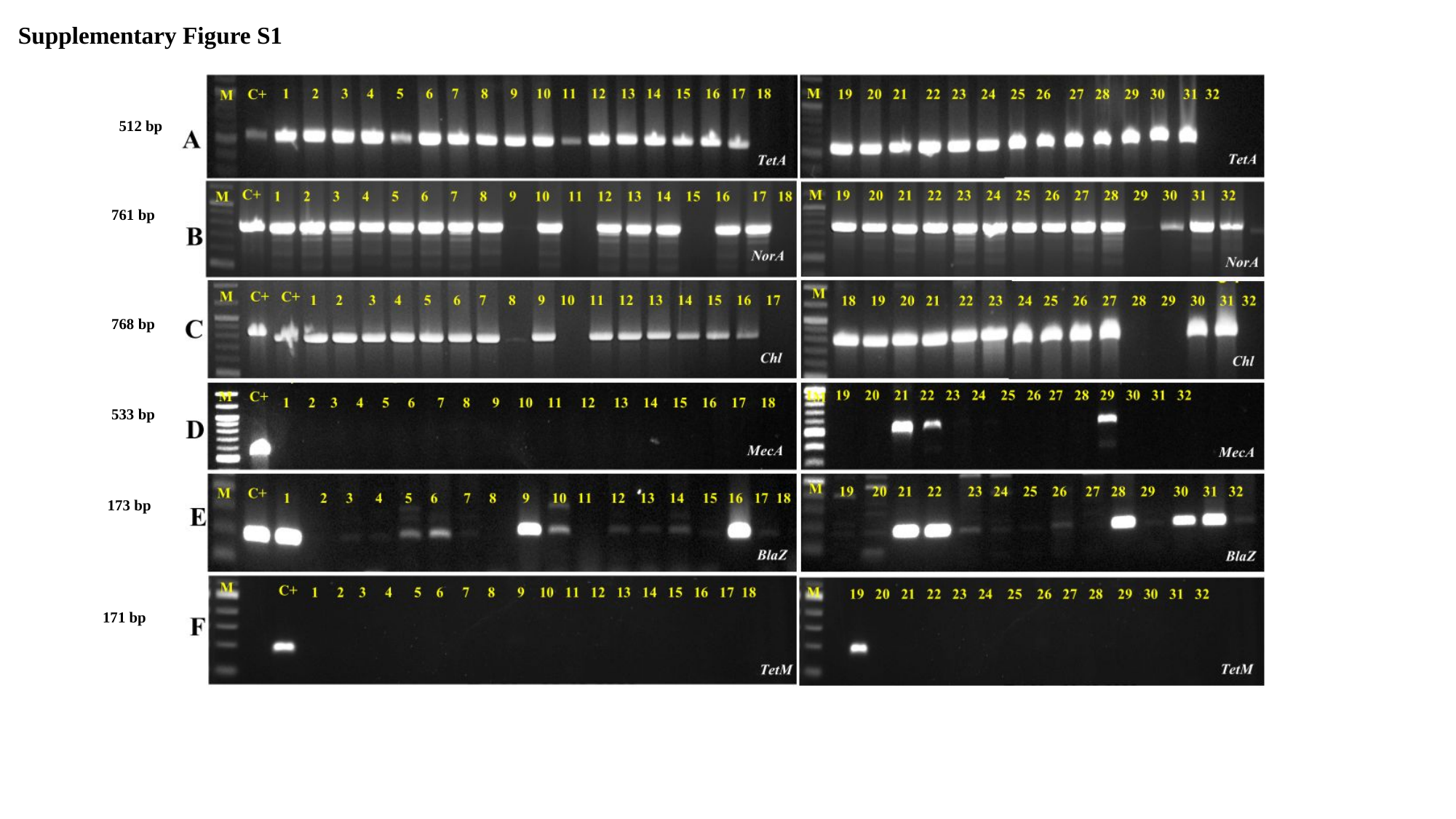

Supplementary Figure S1
512 bp
761 bp
768 bp
533 bp
173 bp
171 bp

## Slide 2
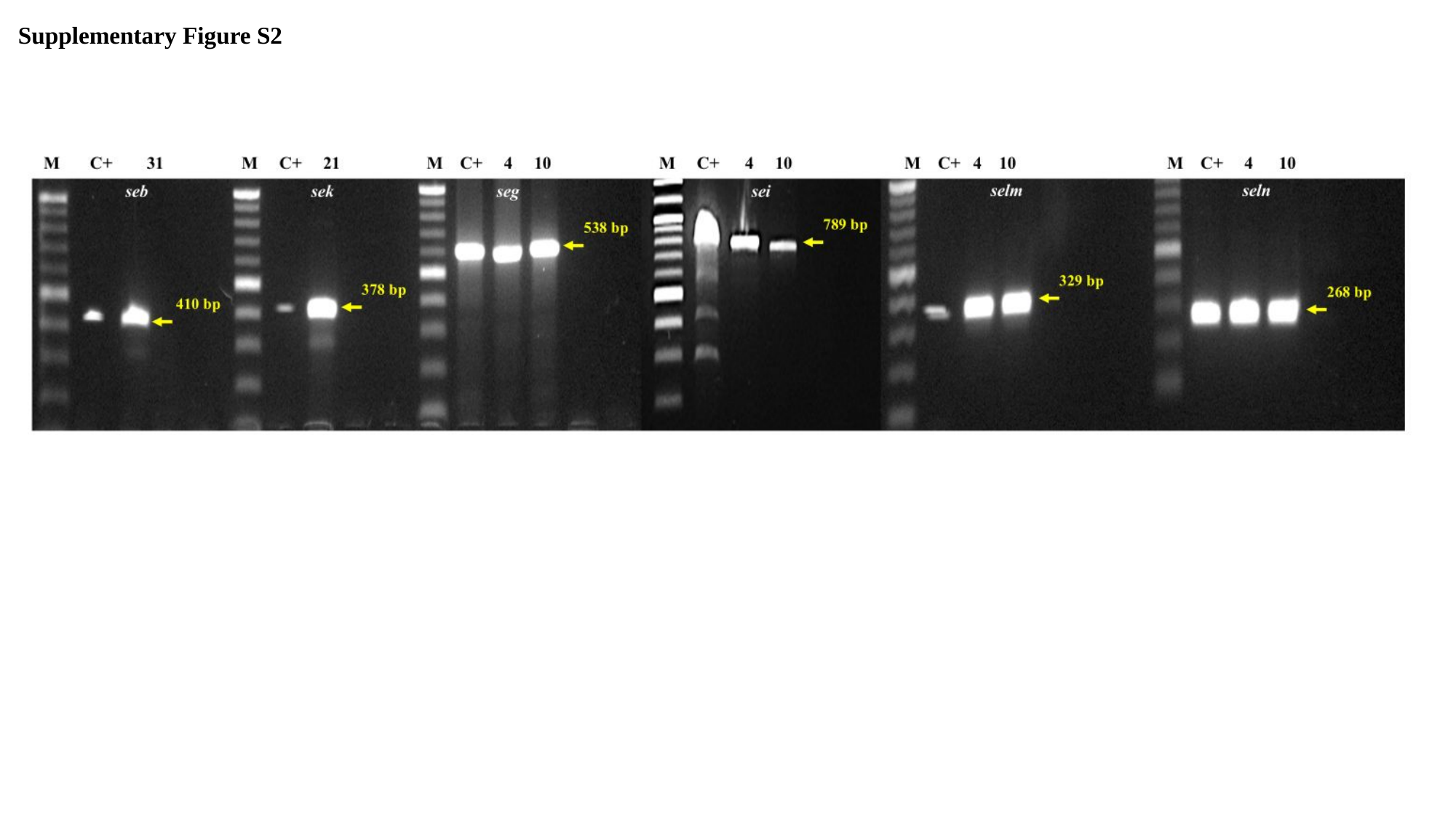

Supplementary Figure S2

Supplement: Supplementary file 1 [file pathogens-14-00107-s001.zip › Supplementary Figures.pptx]
